# Characterization of NDM-1-Producing Carbapenemase in *Proteus mirabilis* among Broilers in China

**DOI:** 10.3390/microorganisms9122443

**Published:** 2021-11-26

**Authors:** Xiaolin Zhu, Yaru Zhang, Zhangqi Shen, Lining Xia, Jinquan Wang, Li Zhao, Ke Wang, Wenhui Wang, Zhihui Hao, Zhihai Liu

**Affiliations:** 1 College of Veterinary Medicine, China Agricultural University, Beijing 100193, China; zhuxiaolin1103@163.com (X.Z.); zhangyaru1@newhope.cn (Y.Z.); szq@cau.edu.cn (Z.S.); wwh6572@163.com (W.W.); 2 College of Chemistry and Pharmaceutical Sciences, Qingdao Agricultural University, Qingdao 266109, China; zhao-li29@163.com (L.Z.); WKPP229329@163.com (K.W.); 3 The New Hope Liuhe Co., Ltd., Qingdao 266061, China; 4 College of Veterinary Medicine, Xinjiang Agricultural University, Urumqi 830052, China; xln750530@163.com (L.X.); wangjinquan163@163.com (J.W.)

**Keywords:** *Proteus mirabilis*, carbapenemase, *bla*_NDM-1_, chicken

## Abstract

Carbapenem-resistant pathogens mediated by metallo-beta-lactamases (MBLs) have spread worldwide, where NDM-1 is a typical and key MBL. Here, we firstly discussed the distribution characterization of NDM-1, which produces multidrug-resistant *Proteus mirabilis* among broilers in China. From January to April 2019, 40 (18.1%, 40/221) *bla*_NDM-1_-carrying *P. mirabilis* strains were recovered from commercial broilers in slaughterhouse B in China. All the isolates were resistant to imipenem, meropenem and other β-lactams. These isolates belong to five clusters identified via pulsed field gel electrophoresis (PFGE). Further studies on twenty representative strains revealed that seven *bla*_NDM-1_ genes were located on plasmids with sizes of 104.5–138.9 kb. Notably, only three strains (PB72, PB96 and PB109) were successfully transferred to *Escherichia coli* J53, while the other four isolates were located in nontransferable plasmids. The rest were harbored in chromosomes. Ulteriorly, based on whole genome sequencing (WGS), these twenty isolates showed four typical phylogenetic clades according to single nucleotide polymorphisms (SNPs) of a core genome and presented four main genomic backbone profiles, in which type II/III strains shared a similar genetic context. All of the above is evidence of *bla*_NDM__-1_ transmission and evolution in *P. mirabilis*, suggesting that the prevalence may be more diverse in broiler farms. Accordingly, as intestinal and environmental symbiotic pathogens, *bla*_NDM-1_-positive *P. mirabilis* will pose greater threats to the environment and public health.

## 1. Introduction

*Proteus mirabilis* is an opportunistic pathogen belonging to the *Enterobacteriaceae* family, widely distributed in the natural environment and the intestines of humans and animals. Clinically, it can cause diarrhea, sepsis, respiratory issues and urinary tract infections, with a potential risk of zoonosis [1,2,3]. Along with *Escherichia coli*, *Salmonella* and methicillin-resistant *Staphylococcus aureus* (MRSA), it is considered to be an important cause of infections in humans and animals [4,5]. *P. mirabilis* is intrinsically resistant to tetracycline, tigecycline and polymyxins [6]. Carbapenems are potent β-lactam antibiotics are still an crucial choice in the treatment of severe human infections because of their low side effects, they are banned for use in veterinary medicine [7]. However, the reports of carbapenem resistant bacteria in livestock, wild animals, food products and the environment have increased [8,9,10,11], which has gradually aroused high levels of attention globally in the fields of human and veterinary clinical and ecological environments. The anxieties surrounding this threat can be illustrated by the increase in carbapenemases, including New Delhi metallo-β-lactamase (NDM), *Klebsiella pneumoniae* carbapenemase (KPC) and carbapenem-hydrolyzing oxacillinase 48 type β-lactamases (OXA-48). As a major type of carbapenemase, NDM can impair the efficacy of almost all β-lactams (except aztreonam), and the therapeutic options for infection are mostly limited to polymyxins, tigecycline, fosfomycin and cefiderocol [12,13], the idea that NDM may acquire additional gene resistance in *P. mirabilis* is worrisome. Hospitals in many countries have widely reported *bla*_NDM-1_-positive *P. mirabilis* [14,15,16,17], but only sporadic reports have been published regarding animals used as food sources [18]. Here, we describe a short-term slaughterhouse outbreak caused by *bla*_NDM-1_-positive *P. mirabilis* and perform microbiological characterization of *bla*_NDM-1_-positive *P. mirabilis*.

## 2. Materials and Methods

### 2.1. Bacterial Isolation, Species Identification and Detection of β-Lactamases Genes

From January to April 2019, a total of 556 samples were collected from four chicken farms (*n* = 212, cloacal swab) and two slaughterhouses (*n* = 344, caecum contents) in Shandong Province, China (Table S1). Cloacal samples were randomly collected from four farms (farm 1:40; 2:72; 3:40 and 4:60) that contained 15,000–20,000 broilers per farm; caecum samples were randomly collected from two slaughterhouses (slaughterhouse A:123; B:221) that contained 20,000–30,000 broilers per slaughterhouse. For the isolation of carbapenem-resistant *P. mirabilis* (CRP), swabs were cultured in 2 mL of brain heart infusion (BHI) broth at 37 °C for 5–6 h, and the cecum was cut directly in a 10 mL centrifuge tube, followed by streaking on salmonella shigella (SS) agar plates (Luqiao, Beijing, China) containing 1 mg/L of imipenem and 30 mg/L of vancomycin (Baomanbio, Shanghai, China) and incubated at 37 °C for 18–24 h. The morphologically representative clones with black centers were isolated and boiled to extract DNA and were confirmed to be *P. mirabilis* via 16S rDNA sequencing [19]. All the strains were sub-cultured at least twice prior to further investigation. Common β-lactamase genes, including carbapenemases (*bla*_NDM_, *bla*_KPC_, *bla*_VIM_, *bla*_IMP_ and *bla*_OXA-48_) and extended-spectrum β-lactamases (ESBLs) (*bla*_CTX-M_, *bla*_TEM_, *bla*_SHV_, *bla*_OXA-1_, *bla*_OXA-2_ and *bla*_OXA-10_), were amplified via polymerase chain reaction (PCR) for all the strains; specific primers were used with 2 × Taq Master Mix (Dye Plus) (Vazyme Biotech, Nanjing, China), and the carbapenemase-positive products were sequenced (Table S2). The sequences were compared with the reported sequences from the GenBank nucleotide database at http://www.ncbi.nlm.nih.gov/blast/ (accessed on 23 November 2021). Fifty *P. mirabilis* strains without carbapenemase were randomly selected for use as *bla*_NDM-1_-negative control strains.

### 2.2. Antimicrobial Susceptibility Testing

The agar dilution method was used to determine the MICs of *bla*_NDM-1_-positive *P. mirabilis*, and their transconjugants were screened with sodium-azide-resistant *E. coli* J53 against 11 antibiotics. The results were interpreted in accordance with the EUCAST guidelines [20]. Reference strain *E. coli* ATCC 25922 served as a quality control.

### 2.3. Pulsed Field Gel Electrophoresis (PFGE)

Genotyping of CRP harboring *bla*_NDM-1_ was performed via PFGE. Chromosomal DNA was digested with 50U of *SmaI* (Takara, Dalian, China) for 3 h at 30 °C. Genomic DNA was separated by CHEF DRIII (Bio-Rad, Hercules, CA, USA) gel electrophoresis in a 1% agarose gel in 0.5 × TBE at 14 °C. The operating conditions are as follows: voltage, 6 V/cm; switch angle, 120°; switch time, 5–20 s for 19 h. *Xba*I-digested *Salmonella Braenderup* H9812 was used as the size ladder. The gels were stained with ethidium bromide (Macklin, Shanghai, China), digitally photographed with Gel Doc^TM^ XR+ (Bio-Rad, Hercules, CA, USA) and normalized as TIFF images. The DNA patterns were analyzed using BioNumerics software v7.6 (Applied Maths, Kortrijk, Belgium) to establish a dendrogram of strain relationships. UPGMA was used as a clustering algorithm and the Dice correlation coefficient was used with a 1.2% position tolerance. The strains with more than 85% similarity were considered to be related.

### 2.4. S1-PFGE and Southern Hybridization

The localization of *bla*_NDM-1_ on the plasmids was verified using S1-PFGE and Southern blotting. the DNA of the strain embedded in Seakem Gold gel with S1 nuclease (TakaRa, Dalian, China) was digested and separated via PFGE, with a conversion time of 2.16–63.8 s for 16.5 h. The DNA fragments were transferred to Hybond N+ (GE AMersham, Pittsburgh, PA, USA) and hybridized with DIG-labeled *bla*_NDM-1_ probes. NBT/BCIP was used for color detection (Roche Applied Sciences, Basel, Schweiz).

### 2.5. Conjugation Assay

The transferability of *bla*_NDM-1_ was determined using a membrane filter method and *E. coli* J53 (sodium-azide resistant) as the recipient. Overnight cultures of the donor strain and recipient *E. coli* J53 were cultured in BHI broth at 37 °C and adjusted to 0.5 McFarland standard. The donor strain (30 μL) was then separately conjugated with *E. coli* J53 (90 μL) on a microporous membrane. The conjugation mixtures were then diluted and plated onto selective BHI agar plates supplemented with meropenem (2 mg/L) and sodium azide (200 mg/L) to recover transconjugants. PCR and MICs were performed to confirm that transconjugants were derivatives of the recipient strain *E. coli* J53. The transfer frequencies were calculated as described in a previous report [21].

### 2.6. Whole Genome Sequencing (WGS) and Bioinformatics Analysis

Twenty *bla*_NDM-1_-positive representative strains were randomly selected from the five clonal types shown by PFGE cluster analysis and were further investigated using whole genome sequencing, including clone A (3/8), clone B (3/3), clone C (4/5), clone D (1/1) and clone E (9/23). Genomic DNA were prepared using the TIANamp Bacteria DNA Kit (Tiangen, Beijing, China) and subjected to WGS using the Illumina HiSeq 2500 platform (Novogene Biotech, Beijing, China). Sequencing was performed using a 2 × 150 bp paired-end configuration with a minimal coverage of 50×. After the original sequencing data were obtained, the raw reads were filtered for quality control to remove low-quality reads, adapter and duplication pollution, so as to obtain high-quality clean reads. The reads were assembled utilizing SPAdes v3.10.0 (http://cab.spbu.ru/software/spades/, accessed on 23 November 2021). Initial genome annotations were performed using Rapid Annotation using Subsystem Technology (RAST) (https://rast.nmpdr.org/, accessed on 23 November 2021), Center for Genomic Epidemiology (CGE) services (https://cge.cbs.dtu.dk/services/, accessed on 23 November 2021) and Pathosystems Resource Integration Center (PATRIC) (https://www.patricbrc.org/job/, accessed on 23 November 2021), and the results were manually curated. Insertion elements (IS) and antimicrobial resistance genes were identified using IS Finder and Res Finder 3.2, respectively. Based on this information, the putative coding sequence for the flanking region of the *bla*_NDM-1_ gene was obtained for genetic environmental analysis. Whole genome sequences were used to establish a phylogenetic tree using Parsnp in the Harvest package, which was visualized using figuretree.

## 3. Results

### 3.1. Isolation of Strains and Detection of β-Lactamase Genotype

**Figure 1:**
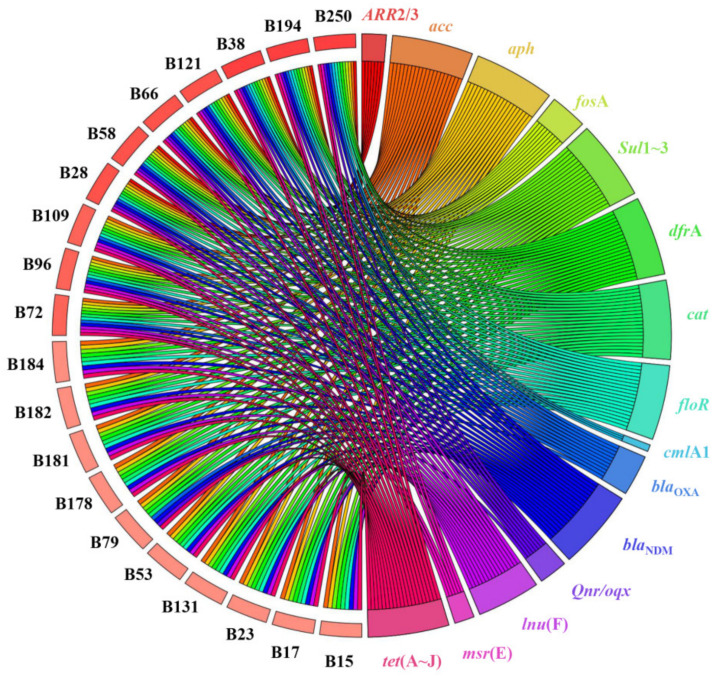
Antibiotic resistance genes in 20 *bla*_NDM-1_-positive *P. mirabilis.* A circos representation showing the presence of antibiotics resistance genes (ARGs) (color) across isolates (black).

In our study, a total of 267 (48.0%) *P. mirabilis* were recovered from 556 samples collected from chicken farms and slaughterhouses. CRP (7.2%, 40/556) were only isolated from slaughterhouse B. PCRs and DNA sequence analysis indicated that all the CRP isolates produced *bla*_NDM-1_. These strains were also found to carry other genes that confer resistance to β-lactams, with 11 strains carrying *bla*_TEM_ (27.5%, 11/40) and *bla*_OXA-1_ (27.5%, 11/40) (unfortunately, the *bla*_TEM_ typing was unsuccessful) and 5 isolates carrying *bla*_OXA-10_ (12.5%, 5/40). No other genes that confer carbapenem resistance, including *bla*_KPC_, *bla*_VIM_, *bla*_IMP_ and *bla*_OXA-48_, were identified in any of the isolates; additionally, β-lactamase genes *bla*_CTX-M_, *bla*_OXA-2_ and *bla*_SHV_ were also not detected in this survey. Twenty of these isolates were successfully sequenced via WGS, and the annotation data further confirmed that these harbored multi-resistant genes, as shown in the circos plot in Figure 1. Compared with *bla*_NDM-1_-positive strains, the detection rates of *bla*_CTX-M_ (70.0%, 35/50), *bla*_OXA-1_ (70.0%, 35/50) and *bla*_OXA-10_ (20.0%, 10/50) were increased, but *bla*_TEM-1_ (14.0%, 7/50) decreased in *bla*_NDM-1_-negative strains; the differences were statistically significant (*p* < 0.05). Subsequently, 29 of 35 *bla*_CTX-M_ were successfully typed, including *bla*_CTX-M-65_ (*n* = 21), *bla*_CTX-M-14_ (*n* = 5), *bla*_CTX-M-27_ (*n* = 2), and *bla*_CTX-M-87_ (*n* = 1), where *bla*_CTX-M-65_ was the predominant subtype.

### 3.2. Antimicrobial Susceptibility

**Figure 2:**
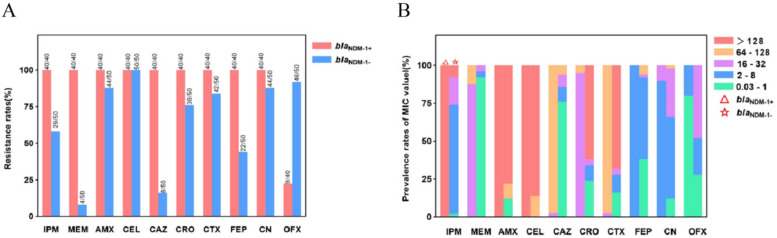
Detection of *bla*_NDM-1_-positive (*n* = 40) and *bla*_NDM-1_-negative (*n* = 50) *P. mirabilis.* (**A**) Agar dilution method of antimicrobial susceptibility test to detect the resistance rates of 10 common antibiotics of *bla*_NDM-1_-positive and -negative strains. (**B**) Proportion of *bla*_NDM-1_-positive and -negative strains to 10 common antibiotics’ MIC distribution interval. △: *bla*_NDM-1+_; ☆: *bla*_NDM-1-_.

Antimicrobial susceptibility results for the forty *P. mirabilis* isolates are listed in Table 1. All the *bla*_NDM-1_-positive *P. mirabilis* exhibited high MICs for imipenem and meropenem (MICs of 128 and 32–64 mg/L, respectively). All isolates were also resistant to amoxicillin, cephalexin, ceftazidime, ceftriaxone, cefotaxime, cefepime and gentamicin (Figure S1) and sensitive to aztreonam. The aminoglycoside antibiotic gentamicin showed a low resistance level, with an MIC value of 4–8 mg/L, and the resistance rate to the quinolone antibiotic ofloxacin was 22.5%, with an MIC distribution of 1–8 mg/L. Overall, nearly a quarter of the forty strains were found to be pandrug resistant to ten antibiotics. Compared with *bla*_NDM-1_-positive strains, most of the antibiotic resistance rates of *bla*_NDM-1_-negative strains dropped from 88.0% to 8.0%, except for the increase in cefalexin and ofloxacin resistance (22.5%→92.0%), while the resistance rates of imipenem and meropenem decreased to 58.0% and 8.0%, respectively (Figure 2A). Of note, three aztreonam-resistant (0→6.0%) strains were found. According to the level of resistance, the MIC values were divided into five intervals. In *bla*_NDM-1_-positive strains, the MIC values of the antibiotics imipenem, amoxicillin, cephalexin, ceftazidime and cefotaxime were found to be >64 mg/L, those of meropenem and ceftriaxone were 16–32 mg/L and those of cefepime, gentamycin and ofloxacin were 0.03-8 mg/L. However, in *bla*_NDM-1_-negative strains, the MIC values of the antibiotics amoxicillin, cephalexin, ceftriaxone and cefotaxime were >64 mg/L, and those of the remaining antibiotics ranged from 0.03 to 8 mg/L (Figure 2B). Most *bla*_NDM-1_-negative strains had lower MIC values and larger distribution spans than *bla*_NDM-1_-positive strains. Of note, the MIC values of ceftriaxone, cefotaxime and ofloxacin were obviously higher than *bla*_NDM-1_-positive *P. mirabilis*.

### 3.3. PFGE Typing and Phylogenetic Analysis

**Figure 3:**
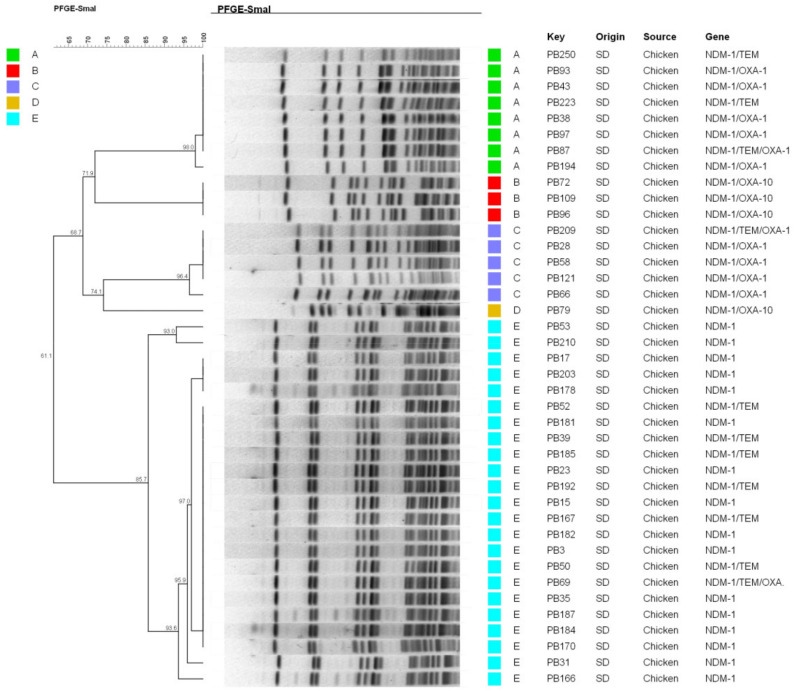
DNA fingerprinting of 40 *bla*_NDM-1_-positive *P. mirabilis* via pulsed field gel electrophoresis (PFGE). The Dice correlation coefficient was set with a 1.2% position tolerance to analyze the similarities of the band type; >85% similarity strains were considered clone related.

**Figure 4:**
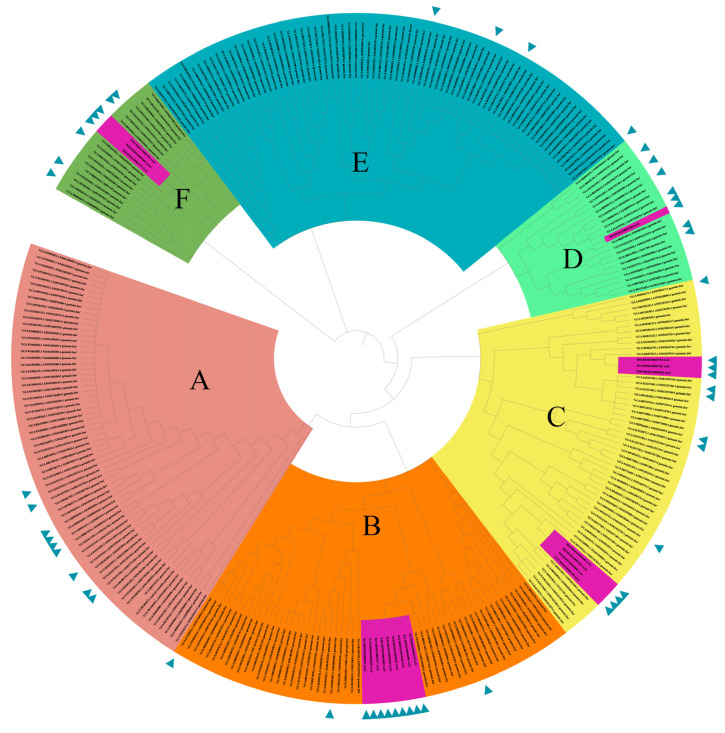
Phylogenetic tree of 298 *bla*_NDM-1_-positive *P. mirabilis* by core genome single nucleotide polymorphisms (SNPs). Twenty isolates shaded by violet were from this study, and 278 genomic data were download from the National Center for Biotechnology Information (NCBI). Different clusters were tagged and shaded different colors. The genome labeled with left triangle were collected from animals (54 in total), while others were from humans. Based on phylogenetic results of whole genome sequencing (WGS), 298 *bla*_NDM-1_ *P. mirabilis* were clustered in 6 major clades: A, B, C, D, E and F, labeled by different color backgrounds. In our study, the 20 *bla*_NDM-1_ *P. mirabilis* isolates colored by purple background were clustered into 4 main clades: B, C, D and F, in which clade B was predominant.

The results of the PFGE cluster analysis showed that forty CRP isolates belonged to five clones, identified as clone A (*n* = 8), clone B (*n* = 3), clone C (*n* = 5), clone D (*n* = 1) and clone E (*n* = 23). Clone E was the dominant clone, with high epidemic and vertical transmission (Figure 3). In addition, CRP isolates of the same cluster were shown to have the same antimicrobial susceptibility patterns. Furthermore, a phylogenetic tree was established based on the core genome of *bla*_NDM-1_-positive *P. mirabilis* by single nucleotide polymorphism (SNP), including the 20 representative isolates in our study, shown with a purple background in Figure 4, and 278 genomes downloaded from the National Center for Biotechnology Information (NCBI) database and almost all available *bla*_NDM_-positive *P. mirabilis* genome data. Thereinto, data on 54 genomes were from animals, and these included the 20 genomes in this study, which can be seen in Figure 4. Six (A, B, C, D, E and F) main phylogenetic branches were observed in all *bla*_NDM-1_-positive *P. mirabilis*, including four major clades and two small groups, F and D. In this study, we found that 20 isolates showed diversity and broad phylogeny and belonged to the branches of B, C, D and F. Although the branches of A and E are the most prevalent phylogeny types worldwide, none of the 20 strains belonged to either of these types. Among all clades, nine isolates shared the same group types, even without subtype branches, and belonged to the majorly prevalent types.

### 3.4. Transfer and Location of the bla_NDM-1_ Gene

**Figure 5:**
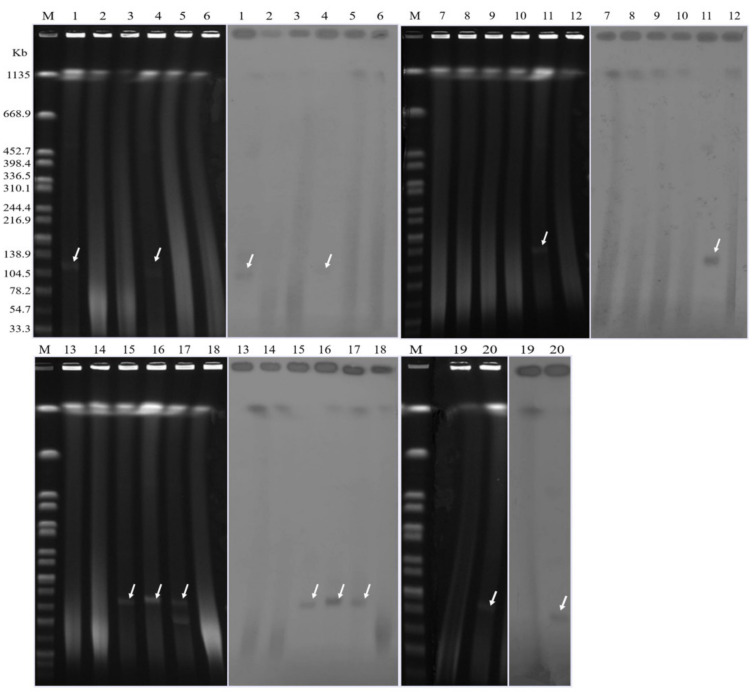
Plasmid analysis revealed by S1-PFGE and Southern hybridization with *bla*_NDM-1_ probe. The arrows represent positive signals of *bla*_NDM-1_ localized on plasmids. Lanes: M (marker, *Salmonella Braenderup* H9812); 1, PB72; 4, PB28; 11, PB109; 15-17, PB121, PB96, PB79; 20, PB58.

Twenty representative CRP strains with different clonal types were selected for S1-PFGE and Southern blotting, including three clone A, three clone B, four clone C, one clone D and nine clone E strains. The results demonstrated that the seven *bla*_NDM-1_ strains were located on similar-size plasmids, ranging from 104.5 to 138.9 kb (Figure 5). Conjugation experiments confirmed that *bla*_NDM-1_ can be transferred into *E. coli* J53. The transconjugants (tPB72, tPB96, tPB109) were successfully recovered from three donor strains with transfer frequencies of 1.3 × 10^−6^–5.8 × 10^−13^ (Table S3). PCR detection of resistance genes confirmed that the transconjugants carried *bla*_NDM-1_. All these transconjugants exhibited similar resistance to β-lactam and aminoglycoside antibiotics to donor strains, including imipenem (MICs 16–128 mg/L), meropenem (MICs 4–32 mg/L), amoxicillin and cephalexin (MICs ≥ 256 mg/L), ceftazidime (MICs ≥ 64 mg/L), ceftriaxone (MICs 32–128 mg/L), cefotaxime (MICs 64–128 mg/L) and cefepime (MICs 2–8 mg/L), but they remained susceptible to ofloxacin and aztreonam. The *bla*_NDM-1_ genes of most other strains were located on the chromosomes. It is worth noting that all the parental strains that successfully conjugated belonged to clone B, while the MICs in several β-lactam antibiotics of transconjugants were higher than those for parental strains (Table 2).

### 3.5. Characterization of the bla_NDM-1_ Genetic Environment

**Figure 6:**
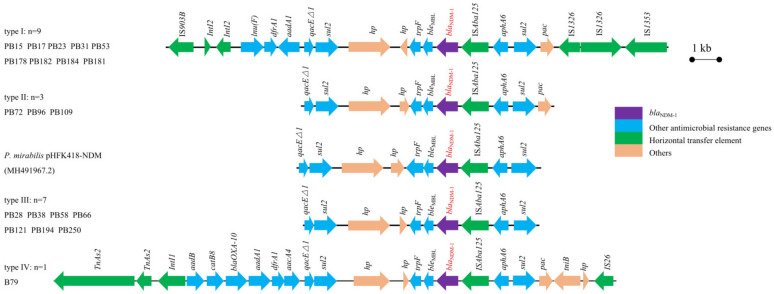
Comparison of different genetic environments of carrying *bla*_NDM-1_ *P. mirabilis*. Arrows indicate the directions of transcription of the genes, and different genes are shown in different colors. The shaded grey areas indicate nucleotide identity (>99.0%) with the same or inverted orientation.

The analysis of whole genomes of strains revealed the distribution of antibiotic resistance genes and replicon types. These twenty strains were mainly divided into four different types of genetic environment (types: I, *n* = 9; II, *n* = 3; III, *n* = 7; IV, *n* = 1) (Figure 6). In our study, conjugation and WGS analysis strongly suggested that the *bla*_NDM-1_ gene was located on the chromosome in type I strains. Although the genetic environment of type I strains showed certain similarities to that of the other bacterial strains in major and mediate segments, a significant difference was observed in farther boundaries at both ends, as shown in Figure 6. In this context, IS*Aba125* insertion sequences were identified immediately upstream of the *bla*_NDM-1_ genes; notably, uncommon insertion element IS*1353,* IS*1326, pac*, *sul2* and *aphA6* were also upstream of the *bla*_NDM-1_. Downstream of *bla*_NDM-1_, the *bla*_NDM-1_ companion gene *ble*_MBL_ was flanked in front of *trpF,* followed by *sul2*, *qacE delta 1*, *aadA1*, *dfrA1* and *lnu(F)* and included transfer element IS*903B* and integron *intI2* in the terminal. The type II genetic environment of *bla*_NDM-1_ shows extremely similar regions with Type I (*pac*-*sul2*-*aphA6*-IS*Aba125*-*bla*_NDM-1_-*ble*_MBL_*-trpF*) and is extremely similar to clinically isolated *P. mirabilis* pHFK418-NDM (GenBank accession number MH491967.2). The genetic environment of type III was almost identical to that of type II (99% query coverage and nucleotide identity), but a difference is that the downstream *sul2* gene in PB66 was truncated into two segments (not shown). In type IV, we found that the downstream region of the *bla*_NDM-1_ flanked an island of multiple resistant genes harboring *aadB*, *CatB8*, *bla*_OXA-10_, *aadA1*, *dfrA1*, *aacA4*, *qacE delta1* and *sul2,* followed by *intI1, trpF* and TnAs2. Regions upstream of *bla*_NDM-1_ genes encompass IS*Aba125*, *aphA6*, *pac*, *TniB* and IS*26* insertion sequences.

## 4. Discussion

As is well known, carbapenem-resistant pathogens are a threat to human public health, especially metallo-β-lactamases including NDM, VIM and IMP. Since NDM-1 was first found in New Delhi, more than 41 subtypes of NDM have become available in GenBank (https://www.ncbi.nlm.nih.gov/genbank/, accessed on 23 November 2021). So far, the presence of NDM has been reported in at least 55 countries and regions, and more than 60 species of bacteria producing NDM-1 or variants have been reported in humans, animals, plants, the environment, etc., with major prevalence in *Enterobacteriaceae* [22,23,24,25,26]. The livestock animals used as food sources and their environment have the dominant reservoir of carbapenem-resistant *Enterobacteriaceae* (CRE)-producing NDM, an important NDM spread section, where *E. coli* and *Klebsiella pneumoniae* have been dominant epidemic host species in animals used as food sources [27]. Recently, *bla*_NDM-1_-positive *P. mirabilis* were continuously identified, the population of which is accelerating. Since the first *P. mirabilis* harboring *bla*_NDM-1_ was reported in patients in New Zealand hospitals in 2009 [28], increasing numbers of cases involving *P. mirabilis* NDM producers have been continuously reported in China [29], Brazil [15], Tunisia [14], Austria [30], India [31], Italy and New Zealand [17,28]. However, all these reports were human, clinical cases of *bla*_NDM-1_-positive *P. mirabilis*; reports of cases in animals used as food sources are still lacking. Here, our studies revealed the dissemination of NDM-containing *P. mirabilis* in commercialized broilers, confirming that in poultry, *P. mirabilis* is a reservoir for *bla*_NDM_.

Generally, a reduction in the susceptibility of *P. mirabilis* to imipenem is mostly due to the intrinsic resistance mechanism loss of outer membrane porins, the decreased expression of PBP1a or the decreased affinity of imipenem to PBP2 [32], which showed low-level resistance. Unfortunately, carbapenemase can confer high-level resistance to β-lactam antibiotics (≥32 µg/mL) by hydrolyzing drugs in comparison to these intrinsic resistance mechanisms. In our study, 40 NDM-producing *P. mirabilis* strains were isolated from a slaughterhouse rather than a commercial farm. As far as we know, the chickens in this slaughterhouse may be from one or more commercial farms. Accordingly, the 40 NDM-producing *P. mirabilis* strains may be prevalent in one or more farms. Our study confirmed the phenomenon that all NDM-producing *P. mirabilis* showed a high carbapenem resistance rate and possessed significantly high resistance levels. As the results showed, the obvious high resistance levels for imipenem and meropenem were observed in *bla*_NDM-1_-positive isolates relative to *bla*_NDM-1_-negative strains (Figure 2). Given this result, a conclusion was drawn that *bla*_NDM-1_ takes responsibility for high carbapenem resistance levels in this study. For *bla*_NDM-1_-positive *P. mirabilis*, in addition to ofloxacin, resistance to all tested antibiotics was demonstrated. In comparison, the resistance spectrum of the non-NDM producer was relatively narrow. Most isolates showed susceptibility to meropenem, ceftazidime and cefepime, which suggested all these antibiotics may be a good option for use in the treatment of infection caused by non-NDM-carrying *P. mirabilis*. The resistance to other β-lactams may be due to other β-lactamases being carried, such as OXA and TEM. Pertinently, almost all *bla*_NDM_-positive *P. mirabilis* resistance to all β-lactams antibiotic still retained susceptibility to ofloxacin in contrast to negative isolates. According to these results and analysis, catching one strain and losing anther was observed in antimicrobial resistance (AMR), which may be thanks to the cost of fitness caused by *bla*_NDM_. Stephan et al. showed a considerable cost of fitness was incurred by *bla*_NDM-1_ plasmid [33]. *P. mirabilis*-encoded β-Lactam resistance genes mainly include *bla*_NDM-1_, *bla*_OXA-1_, *bla*_OXA-10_ and *bla*_TEM-1_, which are consistent with the main epidemic types of *Salmonella*, *Enterobacter cloacae* and *Escherichia coli* in livestock (poultry and pigs) reported in China. They have similar resistance gene spectrum, but the resistance phenotype is more serious [34,35,36]. Notably, in our study, the MIC of *bla*_NDM-1_-harboring isolates from animals used as food sources increased 4-8 times more than from the values in human clinical *P. mirabilis* shown in previous studies [14,37], which were similar to the results shown in investigations regarding wild animals [4]. This suggests that the CRP strains in poultry may be more serious than those in humans, which acts as a warning that the carbapenem-resistant *P. mirabilis* in food animals should be given more attention.

The prevalence of *bla*_NDM-1_ in CRE was mainly mediated by clone transmission and plasmid transfer. However, this depended on the host bacteria and gene. For example, ST258 *Klebsiella pneumoniae* and ST131 *E. coli* are considered the major clones associated with carbapenemase genes [38,39], while the spread of *bla*_OXA-48_, *bla*_VIM_ and *bla*_NDM_ mainly occurs via plasmids [40]. In China, the IncX3-type plasmid has been a dominant vector in the rapid spread of *bla*_NDM-1_ among *Enterobacteriaceae* [29]. Interestingly, these two mediators were present in our research and were confirmed via PFGE, conjugation and Southern blotting. PFGE analysis showed five major clusters, where type E was most common, suggesting clonal transmission may be present among them. The clonal spread of *bla*_NDM-1_ was a common phenomenon in CRE, and evidence is strong that clonal expansion is responsible for a substantial portion of transmitted cases [23,41], while it was rare in *P. mirabilis* among animals used as food sources. Usually, the clonal transmission of *bla*_NDM_ was associated with the IS*26* element [42], which was also present in the gene scaffold of these type E isolates. Additionally, there were other PFGE classifications showing *bla*_NDM_ possess broad host bacteria for *P. mirabilis* and implying *bla*_NDM_ may possess good adaptability in *P. mirabilis* [43,44].

Despite the fact that Southern blotting indicated that seven *bla*_NDM-1_ genes were located on plasmids in twenty tested isolates, only three plasmids (isolates PB72, PB96 and PB109) were successfully transferred. As early as 2011, Anaïs Potron et al. demonstrated that *bla*_NDM_-positive plasmids of different incompatibility groups can transfer into *Escherichia coli*, *Klebsiella pneumoniae*, *Salmonella typhimurium* and *Proteus mirabilis* [45]. In our test, the *bla*_NDM-1_-positive plasmids were transferable to *E. coli* from *Proteus mirabilis*, which serves as further evidence that *bla*_NDM-1_ can transfer between *E. coli* and *P. mirabilis* by plasmid. Unfortunately, for these examples, the type of plasmid incompatibility was unsuccessfully determined based on the WGS data. However, *bla*_NDM-1_-harboring PB72, PB96, PB109 possessed similar-size plasmids and the same gene environments (Figure 6). Moreover, these three isolates belonged to the same cluster, which was confirmed via PFGE. Therefore, there was a possibility of clonal spread occurring among these *P. mirabilis*. Nevertheless, these plasmids in different host bacteria resulted in obvious differences in transfer frequency: 5.8 × 10^−13^, 1.3 × 10^−6^ and 6.2 × 10^−8^ for PB72, PB96 and PB109, respectively. It is possible that these plasmids in PB72, PB96 and PB109 may have been different or slightly evolutionary, further exhibited at the special mobile level. Given this, we tended to think that plasmids took the bulk of responsibility for *bla*_NDM-1_ spread among PB72, PB96 and PB109. Despite another four plasmids that contained *bla*_NDM-1_ with the same plasmid size and gene environment as the three abovementioned transferable plasmids, conjunction failed. It is believed that these plasmids may be untransferable among the four *P. mirabilis* strains. Additionally, these four *P. mirabilis* that carried untransferable plasmids were classed into two PFGE types (clone C and D), which was an obvious difference from PB72, PB96 and PB109 (clone B).

Subsequently, WGS was performed in an attempt to obtain detailed information; however, no complete plasmid sequences were assembled. Even so, three of the same plasmids carrying *bla*_NDM-1_ with sizes of 9430 bp were extracted from the WGS of PB72, PB96 and PB109; they shared an 81.23% homology with the pPrY2001 plasmid recovered from *Providencia rettgeri* and *P. mirabilis* (Figure S2) [6,18]. On the other hand, the S1 results showed that *bla*_NDM-1_ seemed to be located on the same size plasmid (about 110 kb), which was similar to the size found in pPrY2001. All of this information indicated that the three *bla*_NDM-1_ may be harbored in an analogous plasmid, which needs to be clarified in future research. So, it is possible that the spread of these plasmids may be affected or regulated by host bacteria and emerging diversity in transferability in the case of the same plasmid. The presence of mobile and transferable plasmids that harbored *bla*_NDM_, as well as a clonal-spread-associated chromosome encoding *bla*_NDM_, was observed in our study. This may imply the evolution of *bla*_NDM_ transmission from chromosome to transferable plasmid has further developed to mobile plasmids in *P. mirabilis*; however, all this conjecture requires further confirmation. Based on the results of transferable plasmid mediating *bla*_NDM_ and broad clonal host *P. mirabilis*, there is no denying that *bla*_NDM_ may be adjusting to *P. mirabilis* and attempting to increase its survival, which would further increase the risk of widespread infection.

The results emphasized the severity of NDM-1-producing *P. mirabilis* infection in poultry and showed that the animal intestine is an ideal hotbed for *Enterobacteriaceae* bacteria and resistant genes, suggesting that animal-derived resistant bacteria and genes may be spread to communities through direct contact, food chains or the environment, posing a potential threat to human health [46]. In addition, in view of the importance and interdependence of bacterial resistance with regard to humans, domestic animals, wild animals, plants and the environment, following the concept of “one health” to solve this problem should be considered [47]. Therefore, the timely detection of the resistance distribution of *P. mirabilis* in animal breeding, meat product processing, clinical patients and the environment can help to control the generation of resistance from the source and curb the spread of resistance in the food chain, which would have a far-reaching impact on the public.

## 5. Conclusions

In conclusion, this study reported an outbreak of infection in a chicken slaughterhouse caused by NDM-producing *P. mirabilis*_._ The diversity in the mediation of *bla*_NDM_ via chromosomes with transfer elements, untransferable plasmid and mobile plasmid not only revealed the complexity of the spread of *bla*_NDM_ but also suggested *bla*_NDM_ increases the risk of widespread *P. mirabilis* infection among animals used as food sources. The reinforcement of the surveillance of resistance in the animal origin system is extremely urgent for curbing the transmission and persistence of NDM-producing *P. mirabilis*.

## Figures and Tables

**Table 1:** Antimicrobial susceptibility patterns of 40 *bla*_NDM-1_-producing *P. mirabilis* isolates.

Antimicrobial Agent	MIC (mg/L)						
	CloneA (*n* = 8)	CloneB (*n* = 3)	CloneC (*n* = 5)	CloneD (*n* = 1)	CloneE (*n* = 23)	50%	90%
Imipenem	128	128	128	128	128	128	128
Meropenem	32	32	32–64	32	32–64	32	64
Amoxicillin	>256	>256	>256	>256	>256	>256	>256
Cephalexin	>256	>256	>256	>256	>256	>256	>256
Ceftazidime	>64	>64	>64	>64	32->64	>64	>64
Ceftriaxone	16–32	16	16–32	16	16–64	32	32
Cefotaxime	32	32	32	32	32–128	32	32
Cefepime	8	8	8	8	8	8	8
Gentamycin	8	32	8	32	4–8	8	32
Ofloxacin	0.5	2	8	1	0.06	0.06	8
Aztreonam	0.03	0.03	0.03	0.03	0.03	0.03	0.03

**Table 2:** Antibiotic susceptibility profiles of the 3 *bla*_NDM-1_-bearing *P. mirabilis* strains and the corresponding transconjugants to different antibiotics (mg/L).

Strain	MIC (mg/L)										
	IPM	MEM	AMX	CEL	CAZ	CRO	CTX	FEP	CN	OFX	ATM
PB72	128	32	>256	>256	>64	16	32	8	32	2	0.03
PB96	128	32	>256	>256	>64	16	32	8	32	2	0.03
PB109	128	32	>256	>256	>64	16	32	8	32	2	0.03
tPB72J	64	8	>256	>256	>64	128	128	8	32	0.06	0.03
tPB96J	16	4	>256	>256	>64	32	64	2	32	0.06	0.03
tPB109J	128	32	>256	>256	>64	32	64	8	32	0.125	0.03
*E. coli* J53	0.25	0.06	2	16	0.06	64	0.06	0.06	0.06	0.06	0.03

IPM, imipenem; MEM, meropenem; AMX, amoxicillin; CEL, cephalexin; CAZ, ceftazidime; CRO, ceftriaxone; CTX, cefotaxime; FEP, cefepime; CN, gentamycin; OFX, ofloxacin; ATM, aztreonam.

## Data Availability

All data are contained within the article or Supplementary Materials.

## Supplementary Materials

The following are available online at https://www.mdpi.com/article/10.3390/microorganisms9122443/s1, Figure S1: A and B were MDR of *bla*_NDM-1_-positive and negative *P. mirabilis*, respectively. The hotpink and cyan represent resistance and susceptibility, respectively. Figure S2: The blast of pPry2001(GenBank: KF295828.1) plasmid in *Providencia rettgeri* and three same *bla*_NDM-1_-harboring contigs from B109, B72, and B96 genomics in our study. There are 81.23% (7660 bp/9430 bp) homology shared among them. Table S1: Information on the collected samples and numbers of *bla*_NDM-1_-producing *P. mirabilis* strains. Table S2: Primers and PCR amplification conditions for detection of extended-spectrum beta-lactamases and carbapenemases. Table S3: Conjugation frequencies of four *bla*_NDM-1_-bearing *P. mirabilis.*

## References

[1] Gong Z.L., Shi X.L., Bai F., He X.L., Zhang H.Y., Li Y.B., Wan Y., Lin Y.M., Qiu Y.Q., Chen Q.C. (2019). Characterization of a Novel Diarrheagenic Strain of *Proteus mirabilis* Associated with Food Poisoning in China. Front. Microbiol..

[2] Chen C.Y., Chen Y.H., Lu P.L., Lin W.R., Chen T.C., Lin C.Y. (2012). *Proteus mirabilis* urinary tract infection and bacteremia: Risk factors, clinical presentation, and outcomes. J. Microbiol. Immunol. Infect..

[3] Lei C.W., Zhang A.Y., Wang H.N., Liu B.H., Yang L.Q., Yang Y.Q. (2016). Characterization of SXT/R391 Integrative and Conjugative Elements in *Proteus mirabilis* Isolates from Food-Producing Animals in China. Antimicrob. Agents Chemother..

[4] Kang Q., Wang X., Zhao J.N., Liu Z.H., Ji F., Chang H., Yang J.C., Hu S.J., Jia T., Wang X.J. (2020). Multidrug-Resistant *Proteus mirabilis* isolates carrying *bla*_OXA-1_ and *bla*_NDM-1_ from wildlife in China: Increasing public health risk. Integr. Zool..

[5] Gebreyes W.A., Jackwood D., de Oliveira C.J.B., Lee C.W., Hoet A.E., Thakur S. (2020). Molecular Epidemiology of Infectious Zoonotic and Livestock Diseases. Microbiol. Spectr..

[6] Dong D.D., Li M.L., Liu Z.Z., Feng J.T., Jia N., Zhao H., Zhao B.H., Zhou T.T., Zhang X.L.L., Tong Y.G. (2019). Characterization of a NDM-1-Encoding Plasmid pHFK418-NDM From a Clinical *Proteus mirabilis* Isolate Harboring Two Novel Transposons, Tn*6624* and Tn*6625*. Front. Microbiol..

[7] Potter R.F., D’Souza A.W., Dantas G. (2016). The rapid spread of carbapenem-resistant *Enterobacteriaceae*. Drug Resist. Updates.

[8] Guerra B., Fischer J., Helmuth R. (2014). An emerging public health problem: Acquired carbapenemase-producing microorganisms are present in food-producing animals, their environment companion animals and wild birds. Vet. Microbiol..

[9] Köck R., Daniels-Haardt I., Becker K., Mellmann A., Friedrich A.W., Mevius D., Schwarz S., Jurke A. (2018). Carbapenem-resistant Enterobacteriaceae in wildlife, food-producing, and companion animals: A systematic review. Clin. Microbiol. Infect..

[10] Morrison B.J., Rubin J.E. (2015). Carbapenemase producing bacteria in the food supply escaping detection. PLoS ONE.

[11] Hooban B., Joyce A., Fitzhenry K., Chique C., Morris D. (2020). The role of the natural aquatic environment in the dissemination of extended spectrum beta-lactamase and carbapenemase encoding genes: A scoping review. Water Res..

[12] Falagas M.E., Maraki S., Karageorgopoulos D.E., Kastoris A.C., Mavromanolakis E., Samonis G. (2010). Antimicrobial susceptibility of multidrug-resistant (MDR) and extensively drug-resistant (XDR) *Enterobacteriaceae* isolates to fosfomycin. Int. J. Antimicrob. Agents.

[13] Zhanel G.G., Golden A.R., Zelenitsky S., Wiebe K., Lawrence C.K., Adam H.J., Idowu T., Domalaon R., Schweizer F., Zhanel M.A. (2019). Cefiderocol: A Siderophore Cephalosporin with Activity Against Carbapenem-Resistant and Multidrug-Resistant Gram-Negative Bacilli. Drugs.

[14] Kanzari L., Ferjani S., Saidani M., Hamzaoui Z., Jendoubi A., Harbaoui S., Ferjani A., Rehaiem A., Boutiba I.B.B., Slim A. (2018). First report of extensively-drug-resistant *Proteus mirabilis* isolate carrying plasmid-mediated *bla*_NDM-1_ in a Tunisian intensive care unit. Int. J. Antimicrob. Agents.

[15] Ferreira E.F., Beltrão E.M.B., da Silva F.R.F., Alves L.C., Brayner F.A., Veras D.L., Lopes A.C.S. (2019). Association of *bla*_NDM-1_ with *bla*_KPC-2_ and aminoglycoside-modifying enzymes genes among *Klebsiella pneumoniae*, *Proteus mirabilis* and *Serratia marcescens* clinical isolates in Brazil. J. Glob. Antimicrob. Resist..

[16] Aires-de-Sousa M., Ortiz de la Rosa J.M., Goncalves M.L., Costa A., Nordmann P., Poirel L. (2020). Occurrence of NDM-1-producing *Morganella morganii* and *Proteus mirabilis* in a single patient in Portugal: Probable in vivo transfer by conjugation. J. Antimicrob. Chemother..

[17] Bitar I., Mattioni Marchetti V., Mercato A., Nucleo E., Anesi A., Bracco S., Rognoni V., Hrabak J., Migliavacca R. (2020). Complete Genome and Plasmids Sequences of a Clinical *Proteus mirabilis* Isolate Producing Plasmid Mediated NDM-1 from Italy. Microorganisms.

[18] Xie X., Zhang J., Wang H.N., Lei C.W. (2021). Whole genome sequence of a New Delhi metallo-beta-lactamase 1-producing *Proteus mirabilis* isolate SNYG35 from broiler chicken in China. J. Glob. Antimicrob. Resist..

[19] Kim T.W., Kim Y.H., Kim S.E., Lee J.H., Park C.S., Kim H.Y. (2010). Identification and distribution of *Bacillus* species in *doenjang* by whole-cell protein patterns and 16S rRNA gene sequence analysis. J. Microbiol. Biotechnol..

[20] (2020). Version 10.0. Breakpoint Tables for Interpretation of MICs and Zone Diameters. EUCAST. https://www.eucast.org/.

[21] Liu Y.Y., Wang Y., Walsh T.R., Yi L.X., Zhang R., Spencer J., Doi Y.H., Tian G.B., Dong B.l., Huang X.H. (2016). Emergence of plasmid-mediated colistin resistance mechanism MCR-1 in animals and human beings in China: A microbiological and molecular biological study. Lancet Infect. Dis..

[22] Wu W.J., Feng Y., Tang G.M., Qiao F., McNally A., Zong Z.Y. (2019). NDM metallo-β-lactamases and their bacterial producers in health care settings. Clin. Microbiol. Rev..

[23] Politi L., Gartzonika K., Spanakis N., Zarkotou O., Poulou A., Skoura L., Vrioni G., Tsakris A. (2019). Emergence of NDM-1-producing *Klebsiella pneumoniae* in Greece: Evidence of a widespread clonal outbreak. J. Antimicrob. Chemother..

[24] Zhai R.D., Fu B., Shi X.M., Sun C.T., Liu Z.H., Wang S.L., Shen Z.Q., Walsh T.R., Cai C., Wang Y. (2020). Contaminated in-house environment contributes to the persistence and transmission of NDM-producing bacteria in a Chinese poultry farm. Environ. Int..

[25] Wang J., Yao X., Luo J., Lv L.C., Zeng Z.L., Liu J.H. (2018). Emergence of *Escherichia coli* co-producing NDM-1 and KPC-2 carbapenemases from a retail vegetable, China. J. Antimicrob. Chemother..

[26] Cui C.Y., Chen C., Liu B.T., He Q., Wu X.T., Sun R.Y., Zhang Y., Cui Z.H., Guo W.Y., Jia Q.L. (2020). Co-occurrence of Plasmid-Mediated Tigecycline and Carbapenem Resistance in *Acinetobacter* spp. from Waterfowls and Their Neighboring Environment. Antimicrob. Agents Chemother..

[27] Zhang Q.H., Lv L.C., Huang X.Y., Huang Y., Zhuang Z.L., Lu J.X., Liu E.Y., Wan M., Xun H.L., Zhang Z.W. (2019). Rapid Increase in Carbapenemase-Producing *Enterobacteriaceae* in Retail Meat Driven by the Spread of the *bla*_NDM-5_-Carrying IncX3 Plasmid in China from 2016 to 2018. Antimicrob. Agents Chemother..

[28] Williamson D.A., Sidjabat H.E., Freeman J.T., Roberts S.A., Silvey A., Woodhouse R., Mowat E., Dyet K., Paterson D.L., Blackmore T. (2012). Identification and molecular characterisation of New Delhi metallo-β-lactamase-1 (NDM-1)- and NDM-6-producing *Enterobacteriaceae* from New Zealand hospitals. Int. J. Antimicrob. Agents.

[29] Sun L., Xu J., He F. (2019). Genomic characterisation of a *Proteus mirabilis* clinical isolate from China carrying *bla*_NDM-5_ on an IncX3 plasmid. J. Glob. Antimicrob. Resist..

[30] Valentin T., Feierl G., Masoud-Landgraf L., Kohek P., Luxner J., Zarfel G. (2018). *Proteus mirabilis* harboring carbapenemase NDM-5 and ESBL VEB-6 detected in Austria. Diagn. Microbiol. Infect. Dis..

[31] Bhattacharya D., Thamizhmani R., Bhattacharya H., Sayi D.S., Muruganandam N., Roy S., Sugunan A.P. (2013). Emergence of New Delhi metallo-β-lactamase 1 (NDM-1) Producing and Multidrug Resistant Uropathogens Causing Urinary Tract Infections in Andaman Islands, India. Microb. Drug Resist..

[32] Villar H.E., Danel F., Livermore D.M. (1997). Permeability to carbapenems of *Proteus mirabilis* mutants selected for resistance to imipenem or other *β*-lactams. J. Antimicrob. Chemother..

[33] Göttig S., Riedel-Christ S., Saleh A., Kempf V.A.J., Hamprecht A. (2016). Impact of *bla*_NDM-1_ on fitness and pathogenicity of *Escherichia coli* and *Klebsiella pneumoniae*. Int. J. Antimicrob. Agents.

[34] Wang W., Baloch Z., Peng Z.X., Hu Y.J., Xu J., Fanning S., Li F.Q. (2017). Genomic characterization of a large plasmid containing a *bla*_NDM-1_ gene carried on *Salmonella enterica* serovar Indiana C629 isolate from China. BMC Infect. Dis..

[35] Zhu Y., Liu W.Y., Schwarz S., Wang C.Z., Yang Q., Luan T., Wang L.L., Liu S.G., Zhang W.J. (2020). Characterization of a *bla*_NDM-1_-carrying IncHI5 plasmid from *Enterobacter cloacae* complex of food-producing animal origin. J. Antimicrob. Chemother..

[36] Peng Z., Li X.S., Hu Z.Z., Li Z.G., Lv Y.J., Lei M.G., Wu B., Chen H.C., Wang X.R. (2019). Characteristics of Carbapenem-Resistant and Colistin-Resistant *Escherichia coli* Co-Producing NDM-1 and MCR-1 from Pig Farms in China. Microorganisms.

[37] Poirel L., Schrenzel J., Cherkaoui A., Bernabeu S., Renzi G., Nordmann P. (2011). Molecular analysis of NDM-1-producing enterobacterial isolates from Geneva, Switzerland. J. Antimicrob. Chemother..

[38] DeLeo F.R., Chen L., Porcella S.F., Martens C.A., Kobayashi S.D., Porter A.R., Chavda K.D., Jacobs M.R., Mathema B., Olsen R.J. (2014). Molecular dissection of the evolution of carbapenem-resistant multilocus sequence type 258 *Klebsiella pneumoniae*. Proc. Natl. Acad. Sci. USA.

[39] Peirano G., Bradford P.A., Kazmierczak K.M., Badal R.E., Hackel M., Hoban D.J., Pitout J.D.D. (2014). Global Incidence of Carbapenemase-Producing *Escherichia coli* ST131. Emerg. Infect. Dis..

[40] David S., Cohen V., Reuter S., Sheppard A.E., Giani T., Parkhill J., Rossolini G.M., Feil E.J., the European Survey of Carbapenemase-Producing Enterobacteriaceae (EuSCAPE) Working Group, the ESCMID Study Group for Epidemiological Markers (ESGEM) (2020). Integrated chromosomal and plasmid sequence analyses reveal diverse modes of carbapenemase gene spread among *Klebsiella pneumoniae*. Proc. Natl. Acad. Sci. USA.

[41] Duin D.V., Doi Y.H. (2016). The global epidemiology of carbapenemase-producing *Enterobacteriaceae*. Virulence.

[42] Weber R.E., Pietsch M., Frühauf A., Pfeifer Y., Martin M., Luft D., Gatermann S., Pfennigwerth N., Kaase M., Werner G. (2019). IS*26*-Mediated Transfer of *bla*_NDM–1_ as the Main Route of Resistance Transmission During a Polyclonal, Multispecies Outbreak in a German Hospital. Front. Microbiol..

[43] Adamus-Bialek W., Zajac E., Parniewski P., Kaca W. (2013). Comparison of antibiotic resistance patterns in collections of *Escherichia coli* and *Proteus mirabilis* uropathogenic strains. Mol. Biol. Rep..

[44] López C., Ayala J.A., Bonomo R.A., González L.J. (2019). Protein determinants of dissemination and host specificity of metallo-β-lactamases. Nat. Commun..

[45] Potron A., Poirel L., Nordmann P. (2011). Plasmid-mediated transfer of the *bla*_NDM-1_ gene in Gram-negative rods. FEMS Microbiol. Lett..

[46] Sun J., Liao X.P., D’Souza A.W., Boolchandani M., Li S.H., Cheng K., Martínez J.L., Li L., Feng Y.J., Fang L.X. (2020). Environmental remodeling of human gut microbiota and antibiotic resistome in livestock farms. Nat. Commun..

[47] McEwen S.A., Collignon P.J. (2018). Antimicrobial Resistance: A One Health Perspective. Microbiol. Spectr..

