# Supplementary material for: Characterization of NDM-1-Producing Carbapenemase in Proteus mirabilis among Broilers in China
Source: Microorganisms. 2021 Nov 26;9(12):2443. doi: 10.3390/microorganisms9122443 (PMC8707091; doi:10.3390/microorganisms9122443)
Supplement: Supplementary file 1 [file microorganisms-09-02443-s001.zip › Supplementary_material.pdf]

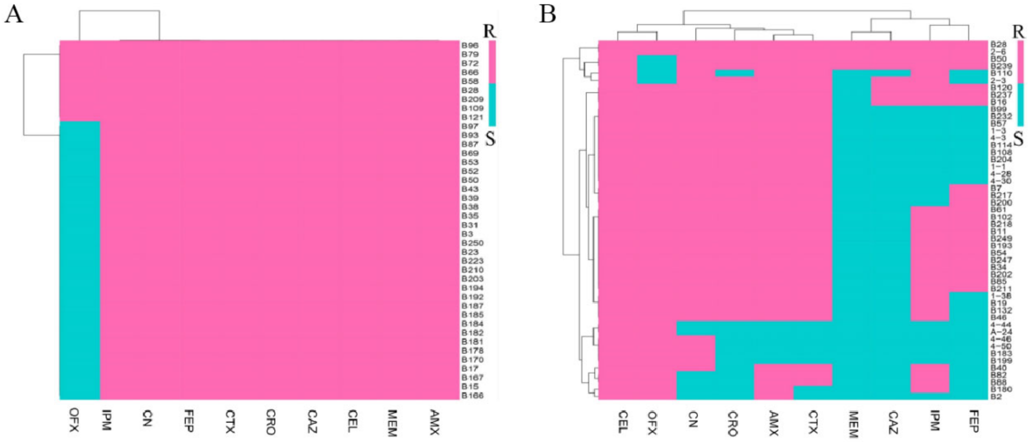

Figure S1. A and B were MDR of *bla*$_{\text{NDM-1}}$-positive and negative *P. mirabilis*, respectively. The hotpink and cyan represent resistance and susceptibility, respectively.

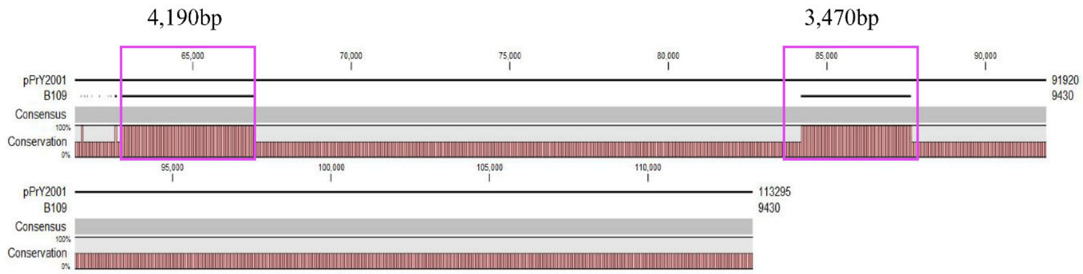

Figure S2. The blast of pPry2001(GenBank: KF295828.1)plasmid in *Providencia rettgeri* and three same *bla*$_{NDM-1}$-harboring contigs from B109, B72, and B96 genomics in our study. There are 81.23% (7660bp/9430bp) homology shared among them.

Table S1. Information on the collected samples and numbers of *bla*$_{NDM-1}$-producing *P. mirabilis* strains.

Sampling sectors		Sample numbers	*P. mirabilis* isolates(%)	*bla*$_{NDM-1}$-producing *P. mirabilis*(%)
Commercial farms	1	40	8(20.0)	0
	2	72	25(34.7)	0
	3	40	0	0
	4	60	42(70.0)	0
Slaughterhouse	A	123	9(7.3)	0
	B	221	183(82.8)	40(18.1)
In total		556	267(48.0)	40(7.2)

Table S2. Primers and PCR amplification conditions for detection of extended-spectrum beta-lactamases and carbapenemases.

Beta-lactamase type		Primer (5′–3′)	Size (bp)
Carbapenemases	NDM-F	CCAATATTATGCACTCTGTCGC	984
	NDM-R	TCAGTGTAGCTTGTCTGCCATGT	
	KPC-F	TGTAAGTTACCGCGCTGAGG	582
	KPC-R	CCAGACGACGGCATAGTCAT	
	VIM-F	GATGGTGTTTGGTCGCATA	390
	VIM-R	CGAATGCGCAGCACCAG	
	IMP-F	TGAGCAAGTTATCTGTATTC	700
	IMP-R	TGAGCAAGTTATCTGTATTC	
	OXA-48-F	GCGTGGTTAAGGATGAACAC	438
	OXA-48-R	CATCAAGTTCAACCCAACCG	
Extended-spectrum beta-lactamases	TEM-F	ATGAGTATTCAACATTTCCGTG	840
	TEM-R	TTACCAATGCTTAATCAGTGAG	
	CTX-M-F	SCSATGTGCAGYACCAGTAA	554
	CTX-M-R	CCGCRATATGRTTGGTGGTG	
	SHV-F	ATGCGTTATATTCGCCTGTG	865
	SHV-R	GTTAGCGTTGCCAGTGCTCG	
	OXA-1-F	TTGAAGGAACTGAAGGTTGT	651
	OXA-1-R	CCAAGTTTCCTGTAAGTGCG	
	OXA-2-F	AAGAAACGCTACTCGCCTGC	600
	OXA-2-R	CCACTCAACCCATCCTACCC	
	OXA-10-F	GTCTTTCGAGTACGGCATTA	720
	OXA-10-R	ATTTTCTTAGCGGCAACTTAC	

Table S3. Conjugation frequencies of four *bla*$_{NDM-1}$-bearing *P. mirabilis*.

	Isolates		
	PB72	PB96	PB109
Conjugation frequency	5.8×10^{-13}	1.3×10^{-06}	6.2×10^{-08}
